# The efficacy and safety of hyoscyamine, dicyclomine, and desipramine in the treatment of irritable pouch syndrome—a retrospective cohort study

**DOI:** 10.1093/crocol/otag063

**Published:** 2026-06-19

**Authors:** Hayden Vaughn, Mille D Long, Edward L Barnes, Hans Herfarth

**Affiliations:** University of North Carolina School of Medicine, Chapel Hill, NC, United States; Division of Gastroenterology and Hepatology, Department of Medicine, University of North Carolina at Chapel Hill, Chapel Hill, NC, United States; Division of Gastroenterology and Hepatology, Department of Medicine, University of North Carolina at Chapel Hill, Chapel Hill, NC, United States; Division of Gastroenterology and Hepatology, Department of Medicine, University of North Carolina at Chapel Hill, Chapel Hill, NC, United States

**Keywords:** ileal pouch-anal anastomosis, pouchitis, irritable bowel syndrome, antispasmodics, antidepressive agents, tricyclic, irritable pouch syndrome

## Abstract

**Background:**

Patients with ileal pouch–anal anastomosis (IPAA) frequently experience high bowel frequency and abdominal pain that are disproportionate to endoscopic inflammation, a condition often termed irritable pouch syndrome. Although antispasmodic agents and neuromodulators are widely used in irritable bowel syndrome, real-world data supporting their effectiveness and durability in patients with pouches remain limited.

**Methods:**

We performed a retrospective cohort study of IPAA patients followed at a pouch clinic between 2014 and 2025. Patients treated with hyoscyamine extended release (ER), dicyclomine, or desipramine for high bowel frequency or abdominal pain were included. Patients could receive multiple therapies sequentially over time. Clinical response was determined by physician-documented symptom improvement. Treatment persistence was assessed using electronic medical record prescription data.

**Results:**

Among 219 eligible patients, 329 treatment courses were analyzed: 143 hyoscyamine ER, 80 dicyclomine, and 106 desipramine. In patients with high bowel frequency, 235 treatment courses resulted in clinical improvement in 68.5% of patients receiving hyoscyamine ER, 65.0% of those receiving dicyclomine, and 63.5% of those receiving desipramine. For abdominal pain (94 treatment courses), improvement for hyoscyamine ER, dicyclomine, and desipramine was observed in 61.5%, 68.3%, and 76.2% of courses, respectively. Approximately 60% of treatment courses persisted beyond one year. Long-term persistence (≥3 years) was highest with dicyclomine (48.7%), followed by desipramine (38.7%) and hyoscyamine ER (37.8%). Adverse events were infrequent and consistent with known safety profiles.

**Conclusions:**

Antispasmodic agents and low-dose neuromodulators are associated with meaningful symptom improvement in patients with irritable pouch syndrome, supporting their role as adjunctive therapies for pouch-related functional symptoms.

## Graphical Abstract

**Graphical Abstract:**
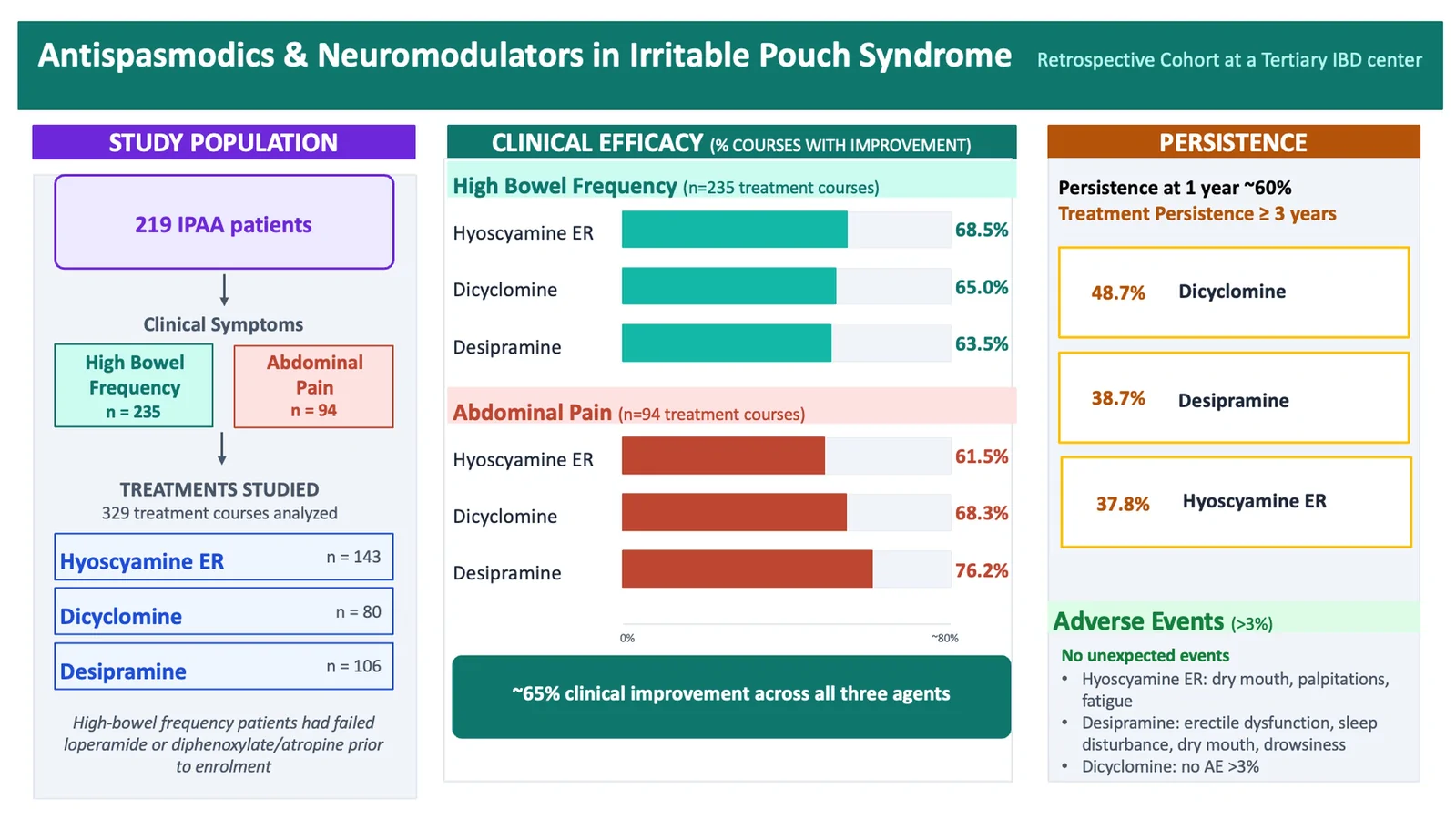

## Introduction

Pouchitis, characterized by endoscopically visible pouch inflammation, is the most common long-term complication after ileal pouch-anal anastomosis (IPAA), affecting up to 80% of patients following colectomy for UC.^1^ The clinical symptoms can include diarrhea, urgency, crampy abdominal pain, fever, bloody bowel movements, dehydration, as well as extraintestinal manifestations such as joint pain. However, in many patients, the severity of clinical symptoms such as bowel frequency, abdominal cramping, and abdominal pain does not correlate with endoscopically visible pouch inflammation or structural pouch abnormalities. Symptoms such as bowel frequency, abdominal cramping, and abdominal pain are also typically observed in patients with irritable bowel syndrome (IBS), a disease entity that is characterized by the absence of structural or inflammatory intestinal pathology. Thus, pouch patients suffering from unexplained high bowel frequency or abdominal pain are similarly classified as having irritable pouch syndrome.^2^ Importantly, irritable pouch syndrome can coexist with other chronic inflammatory conditions of the pouch, such as chronic antibiotic-dependent or antibiotic-refractory pouchitis (ARFP) or Crohn’s-like disease of the pouch.^2^^,^^3^ Similarly, coexistent IBS symptoms have been described in patients with inactive or only mildly active inflammatory ulcerative colitis and Crohn’s disease.^4^^,^^5^ The pathophysiology of these symptoms has been proposed to be similar to IBS patients with an intact and endoscopically and histologically normal gastrointestinal tract, including brain-gut axis dysregulation, altered gut motility, visceral hypersensitivity, microbiome imbalance, stress, and anxiety, and dietary triggers.

While antimotility agents like loperamide or diphenoxylate/atropine are often used as first-line therapy in patients with irritable pouch syndrome and high bowel frequency or abdominal pain, many do not respond to these drugs. Alternative treatments for patients with irritable pouch syndrome include drugs commonly used for diarrhea-predominant IBS or abdominal pain, such as hyoscyamine, dicyclomine, or desipramine.^6^ Overall, irritable pouch syndrome represents a common yet understudied contributor to bowel frequency and abdominal pain following IPAA, often persisting despite minimal or controlled inflammation. Although therapies targeting gut motility and visceral hypersensitivity are routinely extrapolated from studies in IBS, their effectiveness in patients with pouches remains poorly defined. This study provides real-world evidence on the clinical response, safety, and durability of antispasmodic and neuromodulatory therapies in a specialized Multidisciplinary Pouch Clinic.

## Methods

We performed a retrospective cohort study of adult patients who underwent total proctocolectomy with IPAA (J-pouch) and were followed at the University of North Carolina Multidisciplinary Pouch Clinic. Patients were identified through the Carolina Data Warehouse for Health using International Classification of Diseases, Ninth and Tenth Revision, Clinical Modification (ICD-9-CM and ICD-10-CM) codes for pouchitis (569.71 and K91.850), leveraging the NIH-funded Integrating Biology and the Bedside (i2b2) informatics platform, as previously described.^7^^,^^8^ Eligible patients were aged ≥18 years, had undergone IPAA for ulcerative colitis, and received hyoscyamine extended-release formulation (hyoscyamine extended-release [ER]), dicyclomine, or desipramine between January 2014 and July 2025. Endoscopic activity was not uniformly available at the time of treatment initiation and thus was not included in the analyses. Clinical response was determined based on physician-documented assessment of drug efficacy on bowel frequency or abdominal pain following treatment initiation. Persistence was defined as continued prescription refills without documented discontinuation in the electronic medical record. Patients were classified as having antibiotic-responsive pouchitis (ARP), antibiotic-dependent pouchitis (ADP), antibiotic refractory pouchitis (ARFP), or Crohn-like disease of the pouch (CLDP), according to established criteria. Concomitant use of biologics, immunomodulators, or antibiotics within three months before or after the index prescription was recorded. The University of North Carolina Institutional Review Board approved this study.

### Statistical analysis

This study was designed as a descriptive retrospective cohort analysis. Continuous variables are summarized using medians with interquartile ranges (IQRs) or means with standard deviations, as appropriate based on distribution. Categorical variables are presented as counts and percentages.

Clinical efficacy outcomes are reported descriptively as the proportion of treatment courses associated with physician-documented symptomatic improvement for the indication of high bowel frequency or abdominal pain. Because patients could receive more than one study medication sequentially over time, treatment courses—not individual patients—were used as the unit of analysis for efficacy and persistence outcomes. No formal hypothesis testing or comparative statistical analyses between treatment groups were performed, as treatment selection was based on clinical judgment rather than randomization, and the study was not designed or powered to support causal or comparative inference.

Treatment persistence was assessed descriptively using duration categories (<1 year, 1–2 years, and ≥3 years) based on electronic medical record prescription data. Safety outcomes are summarized descriptively as the frequency and proportion of reported adverse events for each therapy. All analyses were performed using SAS statistical software (version 9.4; SAS Institute, Cary, NC, USA).

## Results

### Patient characteristics

From an initial cohort of 752 pouch patients, 219 met all inclusion criteria. Baseline demographic and clinical characteristics are summarized in Table 1. The median age at surgery was 38.6 years (IQR, 27.4–51.5), and the median disease duration before surgery was 6.0 years (IQR, 2.0–12.75). Most patients were male (50.9%) and White (81.7%). Ulcerative colitis was the indication for IPAA in 91.6% of patients.

**Table 1:** Baseline demographic and clinical characteristics of the study cohort (*n* = 219).

	Median	IQR
**Age at surgery, in years**	38.6	27.4–51.5
**Disease duration prior to surgery, in years**	6.0	2.0–12.75
	***n***	**%**
**Male sex**	111	50.9
**Race and ethnicity**		
** White**	179	81.7
** Non-White**	40	18.3
** Hispanic**	8	3.7
**Disease type**		
** Ulcerative colitis**	195	91.6
**Crohn's disease**	15	7.0
** Indeterminate colitis**	3	1.4
Familial Adenomatous Polyposis	4	1.8
** Other**	2	0.9
** Concomitant primary sclerosing cholangitis**	11	5.0
**Disease extent prior to surgery**		
** Proctitis**	3	1.4
** Left-sided colitis**	47	22.0
** Extensive colitis**	90	42.1
** Unknown/FAP**	79	36.1
**IPAA surgical staging**		
** 1**	18	8.2
** 2**	104	47.5
** Modified 2 stage**	7	3.2
** 3**	37	16.9
** Unknown**	53	24.2
**Pouchitis classification**		
** Antibiotic responsive pouchitis**	40	18.3
** Antibiotic dependent pouchitis**	92	42.0
** Antibiotic refractory pouchitis**	31	14.1
** Crohn’s like disease of the pouch**	56	25.6
**Concomitant therapies**		
** Antibiotic**	103	47.0
** Budesonide**	24	11.0
** Anti-Tumor Necrosis Factor (TNF)**	22	10.0
** Vedolizumab**	3	1.4
** Ustekinumab**	10	4.6
** Tofacitinib**	2	0.9
** Thiopurines or methotrexate**	15	6.8

Based on established criteria, 42.0% of patients were classified as having ADP, followed by CLDP (25.6%), ARP (18.3%), and ARFP (14.1%). Nearly half of the cohort (47.0%) were receiving concomitant antibiotics, and 35.0% were treated with topical corticosteroids or advanced immunosuppressive therapies, including biologics or immunomodulators. The median follow-up duration was 70 months (range, 2–145 months).

All patients with high bowel frequency had previously failed treatment with loperamide or diphenoxylate/atropine. Patients could receive more than one study medication sequentially over time; however, due to overlapping side-effect profiles, these agents were generally not prescribed concomitantly. Overall, 329 treatment courses were initiated in 219 patients: 143 with hyoscyamine ER, 80 with dicyclomine, and 106 with desipramine. While 58.4% (128/219) of patients received one study medication, 32.9% (72/219) and 8.7% (19/219) received two or all three agents sequentially during follow-up.

Hyoscyamine ER was typically prescribed twice daily; dicyclomine, 20 mg up to 4 times daily as needed; and desipramine was initiated at 25–50 mg daily, with escalation to 75–100 mg based on response and tolerability. Among patients treated with desipramine, 32.1% (34/106) received 25 mg, 43.4% (46/106) received 50 mg, and 18.9% (20/106) and 5.7% (6/106) received 75 and 100 mg, respectively.

### Clinical efficacy of hyoscyamine ER, dicyclomine, and desipramine in patients with high bowel frequency

For the indication of high bowel frequency refractory to antimotility agents, 235 treatment courses occurred (Table 2). The most prescribed therapies were hyoscyamine ER (55.3%; 130/235) and desipramine (36.2%; 85/235). Clinical improvement was observed in 68.5% (89/130) of hyoscyamine ER treatment courses and 63.5% (54/85) of desipramine treatment courses. Dicyclomine was used less frequently for this indication (8.5%; 20/235) but was associated with clinical improvement in 65.0% (13/20) of cases.

**Table 2:** Clinical improvement by indication and treatment in 219 patients with irritable pouch syndrome.

Indication	Medication	Treatment courses (*n*)	Clinical improvement *n* (%)
**High bowel frequency (*n* = 235)**	Hyoscyamine ER	130	89 (68.5)
	Dicyclomine	20	13 (65.0)
	Desipramine	85	54 (63.5)
**Abdominal pain (*n* = 94)**	Hyoscyamine ER	13	8 (61.5)
	Dicyclomine	60	41 (68.3)
	Desipramine	21	16 (76.2)

Patients could receive more than one study medication sequentially over time, but not concomitantly; therefore, treatment courses rather than individual patients are shown.

### Clinical efficacy of hyoscyamine ER, dicyclomine, and desipramine in patients with abdominal pain

For abdominal pain, 94 treatment courses were identified (Table 2). Dicyclomine was the preferred therapy, accounting for 63.8% (60/94) of treatment courses, with clinical improvement observed in 68.3% (41/60). Hyoscyamine ER and desipramine accounted for 13.8% (13/94) and 22.3% (21/94) of treatment courses, respectively, with improvement rates of 61.5% (8/13) for hyoscyamine ER and 76.2% (16/21) for desipramine.

### Persistence of treatment

Persistence beyond 1 year was observed in a substantial proportion of treatment courses across all three therapies (Table 3). Approximately 35%–40% of patients discontinued therapy within the first year. Dicyclomine demonstrated the highest long-term persistence, with nearly half of patients (48.7%) remaining on an initiated therapy for three years or longer.

**Table 3:** Treatment persistence by medication.

Duration of therapy (years)	Hyoscyamine ER (*n* = 143)	Dicyclomine (*n* = 80)	Desipramine (*n* = 106)
**<1**	57 (39.8%)	29 (36.3%)	39 (36.8%)
**1–2**	32 (22.4%)	12 (15.0%)	26 (24.5%)
**≥3**	54 (37.8%)	39 (48.7%)	41 (38.7%)

Persistence was defined as continued prescription refills without documented discontinuation in the electronic medical record.

### Safety and tolerability

Among patients treated with hyoscyamine ER, the most frequently reported adverse events were dry mouth (11.8%), palpitations (11.8%), and fatigue (3.5%). For desipramine, erectile dysfunction (6.6%), sleep interference (6.6%), dry mouth (3.8%), and drowsiness (3.8%) were the most common adverse events. No specific adverse event reached a frequency greater than 3% in the dicyclomine-treated cohort.

## Discussion

In this large retrospective cohort from a specialized multidisciplinary pouch clinic, we demonstrate that antispasmodic and neuromodulatory therapies commonly used for IBS are associated with clinically meaningful and durable symptomatic improvement in patients with irritable pouch syndrome. Approximately two-thirds of patients treated with extended-release hyoscyamine, dicyclomine, or low-dose desipramine experienced improvement in high bowel frequency or abdominal pain, with substantial treatment persistence extending beyond three years in a significant subset.

These findings are consistent with well-recognized pathophysiological processes in IBS in which symptom generation is driven by altered gut motility, visceral hypersensitivity, and dysregulation of the brain–gut axis, rather than by overt mucosal inflammation, and support the use of anticholinergic agents and tricyclic antidepressants (TCAs).^9^^,^^10^ Importantly, symptom severity in irritable pouch patients—similar to patients with inactive or mildly active inflammatory bowel disease—often correlates poorly with endoscopic or histologic findings, supporting the concept that functional mechanisms contribute substantially to symptom burden.^11^

Desipramine, in particular, has been well studied in functional bowel disorders. Early crossover studies demonstrated significant reductions in stool frequency, abdominal pain, and overall symptoms, especially among patients with diarrhea-predominant IBS.^12^ A more recent multicenter randomized trial confirmed that desipramine can be effective when tolerated, with benefit observed in per-protocol analyses and independent of depressive symptoms.^13^ Notably, subsequent analyses showed no relationship between clinical response and desipramine dose or plasma concentration, supporting the use of low-dose regimens commonly employed in gastroenterology practice.^14^ These findings align closely with the dosing strategies and clinical responses observed in our cohort.

Similarly, antispasmodic agents such as hyoscyamine and dicyclomine have long been used to treat IBS-related pain and bowel frequency, although high-quality long-term data remain limited.^6^^,^^15^ Our study extends this evidence to pouch patients and demonstrates not only short-term symptomatic benefit but also sustained real-world use, suggesting acceptable tolerability and ongoing perceived effectiveness.

The safety profile of these medications was consistent with prior experience in non-pouch populations, with anticholinergic side effects being the most commonly reported for hyoscyamine and dicyclomine, and typical tricyclic-related effects for desipramine.^16–18^ Overall tolerability was acceptable, and no unexpected adverse events were identified, supporting their use in carefully selected patients.

This study has several limitations inherent to its retrospective design, including reliance on physician-documented symptom assessments and the absence of standardized patient-reported outcome measures and endoscopic evaluations at the start of medication use. Residual confounding by concomitant antibiotic and immunosuppressive therapy cannot be fully excluded, as nearly half of the cohort received antibiotics and over one-third received advanced therapies concurrently, which may have independently contributed to symptomatic improvement in some patients. Furthermore, a subset of patients received more than one study medication sequentially, and treatment courses rather than individual patients served as the unit of analysis. This approach does not account for intra-patient clustering or the potential influence of prior treatment experience on subsequent response, which should be considered when interpreting inter-agent comparisons. Treatment persistence as a surrogate for therapeutic durability should be interpreted cautiously, because continued prescription refills may reflect partial symptom benefit, tolerability, the absence of viable alternatives, or physician inertia rather than sustained efficacy. Future prospective studies should incorporate standardized endoscopic assessments at treatment initiation and standardized patient-reported outcome measures to better control for confounding by residual inflammatory activity and to enable more precise, reproducible quantification of symptomatic response to antispasmodic and neuromodulatory therapies in this population.

Nonetheless, strengths of this study include a large, well-characterized pouch cohort, extended follow-up, and consistent management within a dedicated tertiary referral center, offering clinically actionable data to guide therapeutic decision-making in a population for whom prospective trial evidence remains absent.

In summary, antispasmodic agents and low-dose neuromodulators appear to be effective and generally well tolerated for the management of high bowel frequency and abdominal pain in patients with irritable pouch syndrome. Prospective studies incorporating validated symptom indices and objective measures of pouch inflammation are warranted to further define the role of these agents within a structured treatment algorithm for pouch-related functional symptoms.

## Data Availability

The data underlying this study are available from the corresponding author upon reasonable request.

## References

[1] Barnes EL , Herfarth HH, Kappelman MD, et al Incidence, risk factors, and outcomes of pouchitis and pouch-related complications in patients with ulcerative colitis. Clin Gastroenterol Hepatol. 2021;19:1583-1591.e1584. 10.1016/j.cgh.2020.06.035 PMC8552292 32585362

[2] Shen B , Achkar JP, Lashner BA, et al Irritable pouch syndrome: a new category of diagnosis for symptomatic patients with ileal pouch-anal anastomosis. Am J Gastroenterol. 2002;97:972-977. 10.1111/j.1572-0241.2002.05617.x 12003434

[3] Quinn KP , Raffals LE. An update on the medical management of inflammatory pouch complications. Am J Gastroenterol. 2020;115:1439-1450. 10.14309/ajg.0000000000000666 32453044

[4] Wellens J , Sabino J, Vanuytsel T, et al Recent advances in clinical practice: mastering the challenge-managing IBS symptoms in IBD. Gut. 2025;74:312-321. 10.1136/gutjnl-2024-333565 39532478

[5] Colombel JF , Shin A, Gibson PR. AGA clinical practice update on functional gastrointestinal symptoms in patients with inflammatory bowel disease: expert review. Clin Gastroenterol Hepatol. 2019;17:380-390.e381. 10.1016/j.cgh.2018.08.001 PMC6581193 30099108

[6] Lacy BE , Pimentel M, Brenner DM, et al ACG clinical guideline: management of irritable bowel syndrome. Am J Gastroenterol. 2021;116:17-44. 10.14309/ajg.0000000000001036 33315591

[7] Weaver KN , Herfarth HH, Barnes EL. Antibiotic use patterns in the management of chronic pouchitis. Inflamm Bowel Dis. 2022;28:e92-e93. 10.1093/ibd/izab341 PMC9247840 35188204

[8] Weaver KN , Kochar B, Hansen JJ, et al Chronic antibiotic dependent pouchitis is associated with older age at the time of ileal pouch anal anastomosis (J-pouch) surgery. Crohns Colitis 360. 2019;1:otz029. 10.1093/crocol/otz029 PMC6798791 31667470

[9] Trinkley KE , Nahata MC. Treatment of irritable bowel syndrome. J Clin Pharm Ther. 2011;36:275-282. 10.1111/j.1365-2710.2010.01177.x 21545610

[10] Travers P , Lacy BE, Cangemi DJ. Irritable bowel syndrome—less irritable, or better treatments? Curr Opin Gastroenterol. 2024;40:27-33. 10.1097/MOG.0000000000000987 38078610

[11] Kayal M , Bislenghi G, Adamina M, et al ECCO topical review on pouch disorders. J Crohns Colitis. 2025;19:jjaf103. 10.1093/ecco-jcc/jjaf103 40574702

[12] Greenbaum DS , Mayle JE, Vanegeren LE, et al Effects of desipramine on irritable bowel syndrome compared with atropine and placebo. Dig Dis Sci. 1987;32:257-266. 10.1007/BF01297051 3545719

[13] Drossman DA , Toner BB, Whitehead WE, et al Cognitive-behavioral therapy versus education and desipramine versus placebo for moderate to severe functional bowel disorders. Gastroenterology. 2003;125:19-31. 10.1016/s0016-5085(03)00669-3 12851867

[14] Halpert A , Dalton CB, Diamant NE, et al Clinical response to tricyclic antidepressants in functional bowel disorders is not related to dosage. Am J Gastroenterol. 2005;100:664-671. 10.1111/j.1572-0241.2005.30375.x 15743366

[15] Brenner DM , Lacy BE. Antispasmodics for chronic abdominal pain: Analysis of North American treatment options. Am J Gastroenterol. 2021;116:1587-1600. 10.14309/ajg.0000000000001266 PMC8315189 33993133

[16] Mirakhur RK. Anticholinergic drugs. Br J Anaesth. 1979;51:671-679. 10.1093/bja/51.7.671 399194

[17] Page JG , Dirnberger GM. Treatment of the irritable bowel syndrome with bentyl (dicyclomine hydrochloride). J Clin Gastroenterol. 1981;3:153-156. 10.1097/00004836-198106000-00009 7016973

[18] Thiwan S , Drossman DA, Morris CB, et al Not all side effects associated with tricyclic antidepressant therapy are true side effects. Clin Gastroenterol Hepatol. 2009;7:446-451. 10.1016/j.cgh.2008.11.014 PMC2702777 19167522

