# Supplementary material for: The efficacy and safety of hyoscyamine, dicyclomine, and desipramine in the treatment of irritable pouch syndrome—a retrospective cohort study
Source: Crohns Colitis 360. 2026 Jun 19;8(2):otag063. doi: 10.1093/crocol/otag063 (PMC13316432; doi:10.1093/crocol/otag063)
Supplement: otag063_Supplementary_Data [file otag063_supplementary_data.zip › Point to point reply to reviews.docx]

We thank the reviewer for their thoughtful comments. See below a point-to-point reply.

Based on the critiques, we have revised and expanded the limitations section as suggested. Nevertheless, we respectfully submit that this study addresses a genuine gap in the literature: to our knowledge, no prospective data exist to guide antispasmodic and neuromodulatory therapy in this population, and real-world evidence of this scale and clinical granularity retains substantial value for practicing clinicians despite its retrospective nature.

Reviewer 1:

**First, the definition of irritable pouch syndrome is based on symptom documentation without standardized or concurrent assessment of inflammatory activity, raising the possibility of phenotype misclassification, particularly in a cohort with high rates of antibiotic and biologic use.**

Irritable bowel syndrome was defined clinically in routine practice based on clinical symptoms such as increased bowel frequency and severe abdominal pain, which were far beyond the expected normal severity. The patients were all well characterized as antibiotic-responsive pouchitis (ARP), antibiotic-dependent pouchitis (ADP), antibiotic-refractory pouchitis (ARFP), or Crohn-like disease of the pouch (CLDP), according to established criteria that included past endoscopic and clinical course and concurrent therapies , which are outlined in the demographics table. However, pouchoscopies were not systematically performed at the time antispasmodics or neuromodulators were initiated, reflecting real-world decision-making.

We had included this limitation in the discussion, but expanded the limitations section with the following statement: *”**Future prospective studies should incorporate standardized endoscopic assessments at treatment initiation and standardized patient-reported outcomes measures to better control for confounding by residual inflammatory activity and to enable more precise, reproducible quantification of symptomatic response to antispasmodic and neuromodulatory therapies in this population.”*

**Second, the use of treatment courses as the unit of analysis, while pragmatic, introduces potential bias related to intra-patient clustering and treatment sequencing that is not explicitly addressed.**

We agree this is a limitation; a formal mixed-effects model accounting for within-patient clustering would be appropriate in a larger, prospective study, but given the descriptive intent of this analysis, we believe the approach is appropriate and this limitation is now explicitly acknowledged in the limitation section on pages 14- 15. We added:

“*Furthermore, a subset of patients received more than one study medication sequentially, and treatment courses rather than individual patients served as the unit of analysis; this approach does not account for intra-patient clustering or the potential influence of prior treatment experience on subsequent response, which should be considered when interpreting inter-agent comparisons.”*

**Third, the reliance on physician-documented symptom improvement, although reflective of real-world practice, limits precision and may overestimate response compared with validated patient-reported outcomes.**

We agree with this assessment, due to the retrospective character of the study and the absence of standardized PRO assessment at each patient clinic visit, PROs were not available. We added the following limitation in the limitation section on pages 14-15, incorporating the first point of the critique as well.

*”Future prospective studies should incorporate standardized endoscopic assessments at treatment initiation and standardized patient-reported outcomes measures to better control for confounding by residual inflammatory activity and to enable more precise, reproducible quantification of symptomatic response to antispasmodic and neuromodulatory therapies in this population.”*

Additionally, treatment persistence should be interpreted cautiously as a surrogate for durability, as continuation may reflect partial benefit, tolerability, or lack of alternatives rather than sustained efficacy. The potential confounding effects of concomitant therapies—particularly antibiotics and advanced immunosuppression—also warrant more clear acknowledgment.

We agree with this limitation and added the following sentences to the limitation section on pages 14-15.

*“Residual confounding by concomitant antibiotic and immunosuppressive therapy cannot be fully excluded, as nearly half of the cohort received antibiotics and over one-third received advanced therapies concurrently, which may have independently contributed to symptomatic improvement in some patients”*

*“Treatment persistence should be interpreted cautiously as a surrogate for therapeutic durability, because continued prescription refills may reflect partial symptom benefit, tolerability, the absence of viable alternatives, or physician inertia rather than sustained efficacy.”*

Reviewer 2: **Difficult to truly capture Irritable Pouch Syndrome, a diagnosis of exclusion, using retrospective analysis of pouch plus anti-spasmodics or desipramine. Overlap of conditions structural, inflammatory and functional pouch disorders is common and I do think the data here is useful in clinical practice.**

**Is there a way to define the degree of inflammation present in patients who were analyzed and can you show a difference between those with controlled inflammation and persistent pain and increased bowel frequency?**

We agree with this comment, but due to the retrospective design, endoscopic data were not uniformly available. We have added this explicitly to the limitations on pages 14-15.
